# Aging by autodigestion

**DOI:** 10.1371/journal.pone.0312149

**Published:** 2024-10-17

**Authors:** Frank A. DeLano, Geert W. Schmid-Schönbein

**Affiliations:** Shu Chien-Gene Ley Department of Bioengineering, Center for Autodigestion Innovation, University of California San Diego, La Jolla, California, United States of America; Shanghai Jiao Tong University, CHINA

## Abstract

The mechanism that triggers the progressive dysregulation of cell functions, inflammation, and breakdown of tissues during aging is currently unknown. We propose here a previously unknown mechanism due to tissue autodigestion by the digestive enzymes. After synthesis in the pancreas, these powerful enzymes are activated and transported inside the lumen of the small intestine to which they are compartmentalized by the mucin/epithelial barrier. We hypothesize that this barrier leaks active digestive enzymes (e.g. during meals) and leads to their accumulation in tissues outside the gastrointestinal tract. Using immune-histochemistry we provide evidence in young (4 months) and old (24 months) rats for significant accumulation of pancreatic trypsin, elastase, lipase, and amylase in peripheral organs, including liver, lung, heart, kidney, brain, and skin. The mucin layer density on the small intestine barrier is attenuated in the old and trypsin leaks across the tip region of intestinal villi with depleted mucin. The accumulation of digestive enzymes is accompanied in the same tissues of the old by damage to collagen, as detected with collagen fragment hybridizing peptides. We provide evidence that the hyperglycemia in the old is accompanied by proteolytic cleavage of the extracellular domain of the insulin receptor. Blockade of pancreatic trypsin in the old by a two-week oral treatment with a serine protease inhibitor (tranexamic acid) serves to significantly reduce trypsin accumulation in organs outside the intestine, collagen damage, as well as hyperglycemia and insulin receptor cleavage. These results support the hypothesis that the breakdown of tissues in aging is due to autodigestion and a side-effect of the fundamental requirement for digestion.

## Introduction

Aging is accompanied by a loss of numerous cell and tissue functions, clinically manifest co-morbidities with increased susceptibility to diseases, and eventually by full organ failure at the time of death. A spectrum of biological processes (cell and mitochondrial functions, stem cell proliferation and differentiation, genetic lesions, histones, DNA repair mechanisms, epigenetics, protein folding, intra- and inter-cellular signaling, nutrient utilization) become dysregulated, unstable, and exhausted [1]. Vascular and immunological cell functions become impaired with pathological restructuring and development of age-related risk factors and diseases [2]. Different tissues share molecular and cellular mechanisms for micro- and macrovascular pathologies in aging [2,3].

Aging is also accompanied by chronic low-grade markers for inflammation [4,5]. Since the inflammatory cascade fundamentally serves tissue repair [6], a chronic mechanism exists in aging that causes tissue damage. In all organs, the cells and the extracellular matrix are degrading, for which mechanisms due to reactive oxygen species, radiation exposure, and repeat small injuries have been proposed [7–12]. However, none has been universally accepted to explain the source of cell dysfunctions and inflammation in aging.

We postulate here a previously unexplored mechanism for aging due to *autodigestion* [13,14] involving the digestive enzymes. After synthesis in the pancreas, they are discharged into the duodenum and small intestine where they degrade large masses of biomolecules daily. Inside the small intestine, digestive enzymes are concentrated (at a mM level), fully activated, and relatively non-specific to facilitate the breakdown of diverse polymeric food sources into lower molecular weight monomeric-size nutrients. Autodigestion of one’s intestine is primarily prevented by compartmentalizing the digestive enzymes in the lumen of the intestine by the mucin/epithelial barrier [15]. While this barrier is permeable to small molecular weight nutrients (ions, amino acids, monosaccharides, etc.), normally it has a low permeability to larger molecules, such as pancreatic digestive enzymes [16].

During aging, the permeability of the intestinal barrier was reported to shift insignificantly for relatively low-molecular-weight sugars [17] but its properties remain unknown for molecules the size of digestive enzymes. We showed that even during a single meal, the permeability of digestive proteases may increase so that their enzyme activity is detectable in the circulation [18]. Thus, we hypothesize that digestive enzymes leak daily across the mucin-epithelial barrier into tissues and organs outside the pancreas and intestines, where they damage the extracellular matrix and attenuate multiple cell functions characteristic of aging.

Accordingly, we determined in rats of old age the transport of key digestive enzymes (including trypsin, elastase, lipase, and amylase) out of the small intestine past the mucin/epithelial barrier and their accumulation in peripheral organs. Digestive proteases cause multiple forms of tissue damage, including degradation of collagen and cleavage of membrane receptors (e.g. the insulin receptor) [19]. Treatment of old rats for 14 days with a pancreatic trypsin inhibitor (tranexamic acid) [16] attenuates the breakdown of the mucin barrier, reduces the accumulation of digestive enzymes in peripheral organs, collagen degradation, and reduces insulin receptor cleavage and hyperglycemia in old rats.

## Methods

### Animals and tissue collection

Animal protocols were reviewed and approved by the University of California San Diego Institutional Animal Care and Use Committee. Male Wistar rats (Harlan Sprague Dawley Inc., Indianapolis, IN) at maturity (4 months, 300 to 350 gm) and at old age (24 months, 375 to 450 gm) were included in the study. The animals were maintained on standard laboratory chow (8604 Teklad rodent diet; Harlan Laboratories, Indianapolis, Ind) without restriction and water ad libitum and maintained in a separate room without pathogen-free conditions. They were confirmed to exhibit normal mobility, water and food consumption, and fecal material discharge. Animals that exhibited signs of morbidities were excluded. A subgroup of old animals was given a serine protease inhibitor (tranexamic acid, 14 days) in drinking water (137 mM, exchanged daily) which at a minimum fluid consumption of 40 ml/day amounts to a minimum dose of 0.39 gm/kg/day.

A femoral venous catheter was placed after general anesthesia (pentobarbital sodium, 50 mg/kg [Abbott Laboratories, North Chicago, Ill], intramuscularly after local anesthesia [2% lidocaine HCl; Hospira, Inc, Lake Forrest, Ill]). Tissues (intestine, liver, lung, heart, kidney, brain, mesentery, skin) were immediately collected after euthanasia (Beuthanasia i.v., 120 mg/kg, Schering-Plough Animal Health Corp, Union, NJ), fixed (formalin, 10%, neutral buffered, 1 hr), postfixed (fresh formalin, 24 hrs), and stored (formalin, 10%). The period between initial anesthesia and fixation of the tissues was below 60 minutes. All tissues were excised with sharp blades to minimize the stretching of collagen before fixation.

### Tissue sections

Formalin-fixed tissues were cut into sections with a vibratome (thickness 40 μm; Pelco Lancer Vibratome Series 1000). The areas of the section were kept above ~3 mm x ~5 mm to permit analysis of digestive enzyme infiltration over diverse regions within an organ.

To generate thin sections for the intestine, a segment of the upper jejunum was embedded in resin (Araldite; Polysciences, Washington, PA) and cut into 1 μm sections (Ultramicrotome, LKB Ultratome Nova). The resin was removed with Maxwell solution (2 g KOH in 10 ml absolute methyl alcohol + 5 ml propylene oxide) [20], rinsed in tap water, incubated in hydrogen peroxide (4%, 1 minute), rinsed (phosphate buffer), and immunolabeled for trypsin (see below).

### Digestive enzyme immunohistochemistry

To determine on the tissue sections the immunolabel density and distribution of digestive enzymes, the following primary antibodies were used: pancreatic trypsin MoAb (D-1): sc-137077(Santa Cruz); pancreatic elastase (ELA1) polyclonal antibody (Biomatik); pancreatic lipase MoAb (A-3): sc-374612 (Santa Cruz); amylase MoAb (G-10): sc-46657 (Santa Cruz). Primary antibodies were diluted to 1–1.5 μl/1000μl of phosphate-buffered saline. They were followed by secondary antibodies (MP-7601 for anti-rabbit IgG; MP-7602 for anti-mouse IgG; ImmPRESS Excel staining kit peroxidase). Two substrate colors were used, red (ImmPact^TM^ AEC Substrate kit peroxidase, sk-4205; Vector®Laboratories) and brown (ImmPACT^TM^ DAB [3,3’-diaminobenzidine] Substrate kit peroxidase, sk4105; and Vectorstain Elite ABC-HRP Kit, Vector®Laboratories). Sections without primary antibodies served as controls. No counterstain was applied to facilitate quantitative label intensity measurements. The concentrations and exposure of primary and secondary antibodies applied to the sections were adjusted (24 hrs and according to protocol by Vector Laboratories, respectively) to achieve full penetration of the antibodies into the tissue sections. For each tissue, the labeling procedures were standardized among the animal groups to permit a quantitative comparison of label densities between ages and treatment with digital image analysis.

### Whole mount tissue labeling

#### Small intestine

Full-thickness tissue blocks of the wall of the proximal jejunum (3 x 5 mm) were fixed from all sides in 10% formalin. Digestive enzymes were detected with primary and secondary antibodies labeled with 3, 3’-diaminobenzidine (DAB, Peroxidase Substrate Kit, ab64238, ABCAM).

The mucin-containing layer on the epithelial cells of the small intestine was stained using alcian blue (pH 2.5, kt 003; Diagnostic BioSystems, Pleasanton, CA) followed by a rinse in distilled water and mounted on a microscope slide (Vector Mount AQ Aqueous Mounting Medium, Vector Laboratories, Burlington, CA).

To co-label the small intestine for mucin and trypsin, the fixed intestine was immersed in the primary antibody against trypsin and stained with DAB substrate. Thereafter the tissue section was embedded in resin and sectioned into thin (1 μm) sections. The mucin label (alcian blue), was applied to the thin section, coverslipped, and imaged.

## Mesentery

The trypsin distribution in intact mesentery sectors was delineated by biotin/avidin immunolabeling with MoAB (D-1), secondary antibody (anti-mouse IgG, MP-7602, ImmPRESS Excel staining kit peroxidase, Vector®Laboratories) with a brown substrate (ImmPACT DAB sk-4105).

## Collagen damage labeling

To localize molecular level subfailure of collagen with specificity [21], sections were labeled with biotin-conjugated collagen hybridizing peptides (B-CHP) that bind unfolded collagen by triple helix formation. The trimeric CHP are thermally dissociated to monomers before use (80°C for 10 min), the hot CHP solution is quickly cooled to room temperature (by immersion into 4°C water for 15 sec), diluted (1 μl in 1000 μl phosphate buffer saline, applied solution 7.5 mM) and immediately applied to the section (dead time <1 min). In this way, most CHP peptides are expected to remain as active monomers during the staining process, based on kinetic studies on CHP triple helix folding [22]. Sections are incubated overnight at room temperature, and unbound B-CHP is washed (3 times in 1ml of 1xPBS for 30min at room temperature). To visualize the B-CHP, the tissue sections are incubated with streptavidin peroxidase (sk-5704, Vector®Laboratories, according to manufacturer instructions) and then to a substrate (*ImmPact* AEC Substrate Kit Peroxidase; sk-4205, Vector Laboratories) at room temperature (for periods between 1 and 10 min depending on the tissue). The B-CHP label intensity on the sections is recorded by digital brightfield microscopy (40x, numerical aperture 0.5).

## Glucose analysis

At the time of tissue collection, fresh femoral arterial blood was used to measure the blood glucose level (Contour, Bayer Diabetes Care, Tarrytown, NY) and the percent of glycated hemoglobin levels (A1C Home Test; Bristol-Myers-Squibb Co; NY, NY).

### Brain insulin receptor density

Measurements of insulin receptor density were carried out by immunolabeling its extracellular domain on fixed tissue sections (10% formalin, neutral buffered) with a primary antibody (M-20, sc-57344 HRP, monoclonal antibody mapping to the N-terminus, Santa Cruz®Biotech) and visualize with a substrate (ImmPACT AEC Substrate kit peroxidase, SK-4205, Vector Laboratories). Sections without primary antibodies were used as negative controls.

### Digital image analysis

Images of the immunolabel density were recorded at multiple magnifications (between 10x objective, numerical aperture 0.25, and 60x, numerical aperture 1.4). They were recorded under standard light conditions with fixed optical and digital camera settings (Spot Insight GIGABIT camera, Sterling Height) so that the camera serves as a quantitative light intensity meter without pixel intensity saturation. Images were analyzed digitally (Photoshop, Adobe 24.4.1.; spatial resolution of 640x480 pixels).

The red color of the biotin-conjugated collagen hybridizing peptides and the red immune substrates was digitally extracted and their intensity was measured on a B/W scale (1 to 256 digital units between white and black, respectively). The density of the immune substrate label was measured in the form of digital light intensity (I) at a constant incident light intensity (I_o_) without a tissue section.

Insulin receptors’ densities on random tissue sections, labeled with an antibody against the extracellular domain, are digitally recorded by placing an optical window on the cell and determining light intensity at a constant incident light I_o_.

Unless specified otherwise, the mean label density per group (3 animals/group) is determined from the average label density per animal (5 tissue sections/animal, ~10 images/section).

### Statistics

Measurements are summarized as mean ± standard deviation. For comparisons between young and old, an unpaired two-tailed Student’s t-test was used. Analysis of variance (ANOVA) was used to test for differences in outcomes of interest among groups. Results were determined to be significant at p<0.05. Bonferroni’s post hoc multiple comparison test was used to determine the significance between individual groups. To obtain statistically conclusive results, the minimum number of animals was estimated assuming equal variances among groups, α = 0.05 and β = 1–0.9. No animals were excluded from the analysis.

## Results

### Digestive enzyme accumulation in old organs outside the gastrointestinal tract

**Fig 1:**
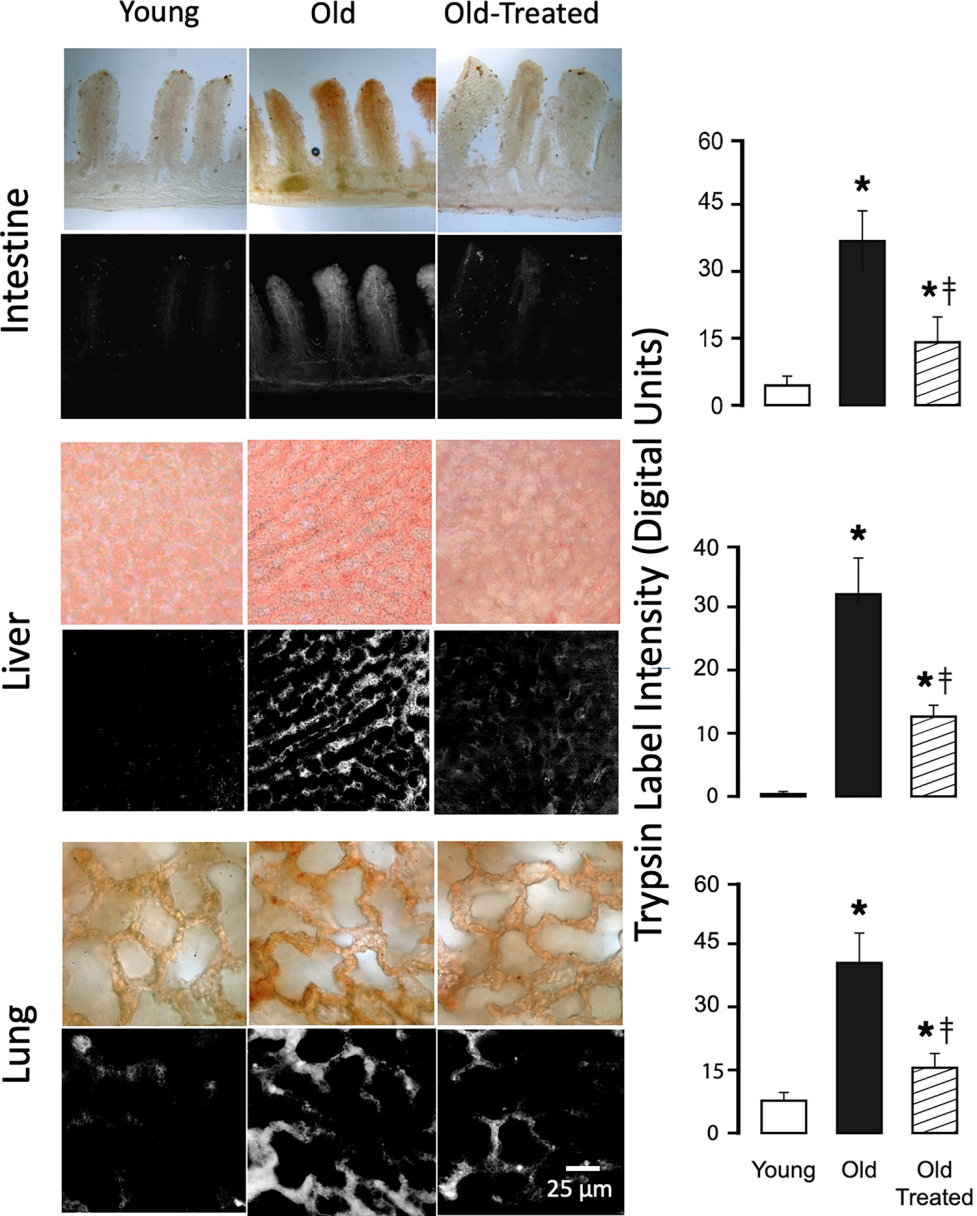
Intestine, liver, and lung in the old, but not the young, are infiltrated by pancreatic trypsin. Pancreatic trypsin label intensity by immunohistochemistry on tissue sections of young (4 months), old (24 months), and old-treated rats (at age 24 months treated with serine protease inhibitor for 14 days) in small intestine, liver, and lung. The tissues are labeled with brown substrate except for the liver (with red substrate). The color images for each organ show the immunohistochemistry labels in the original bright field, and the black/white images show the label density after digital color extraction. The histograms (right column) show the mean ± SD of the trypsin label intensities (digital units). The length scale for all figures is the same. *p<0.05 compared with young group, ‡p<0.05 compared with old untreated rats.

**Fig 2:**
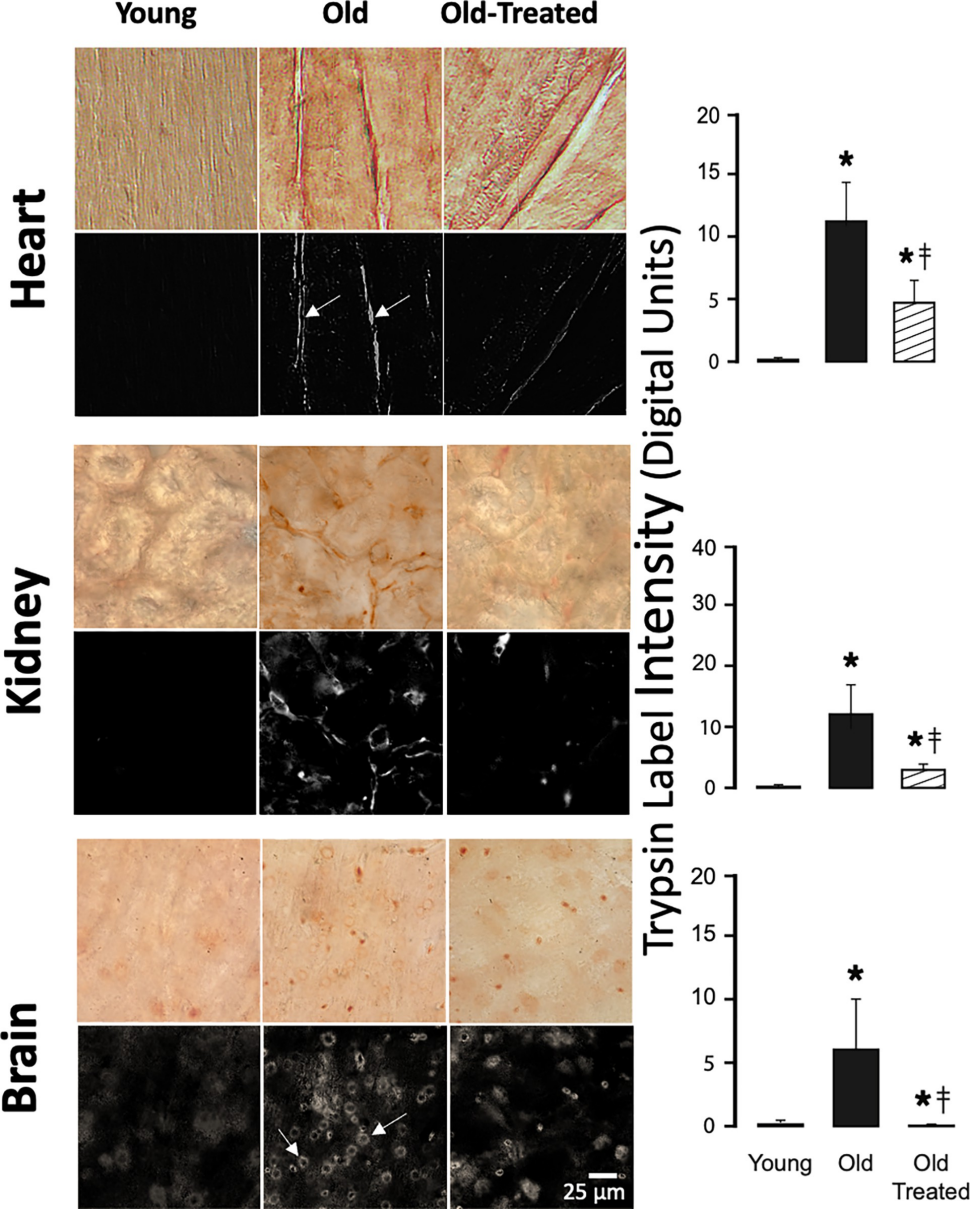
Pancreatic trypsin label density in heart, kidney, and brain is enhanced in the old. Groups, immunohistochemical labeling and measurement techniques, and notation are the same as in Fig 1. The length scale (shown in the brain image) is the same for all images.

**Fig 3:**
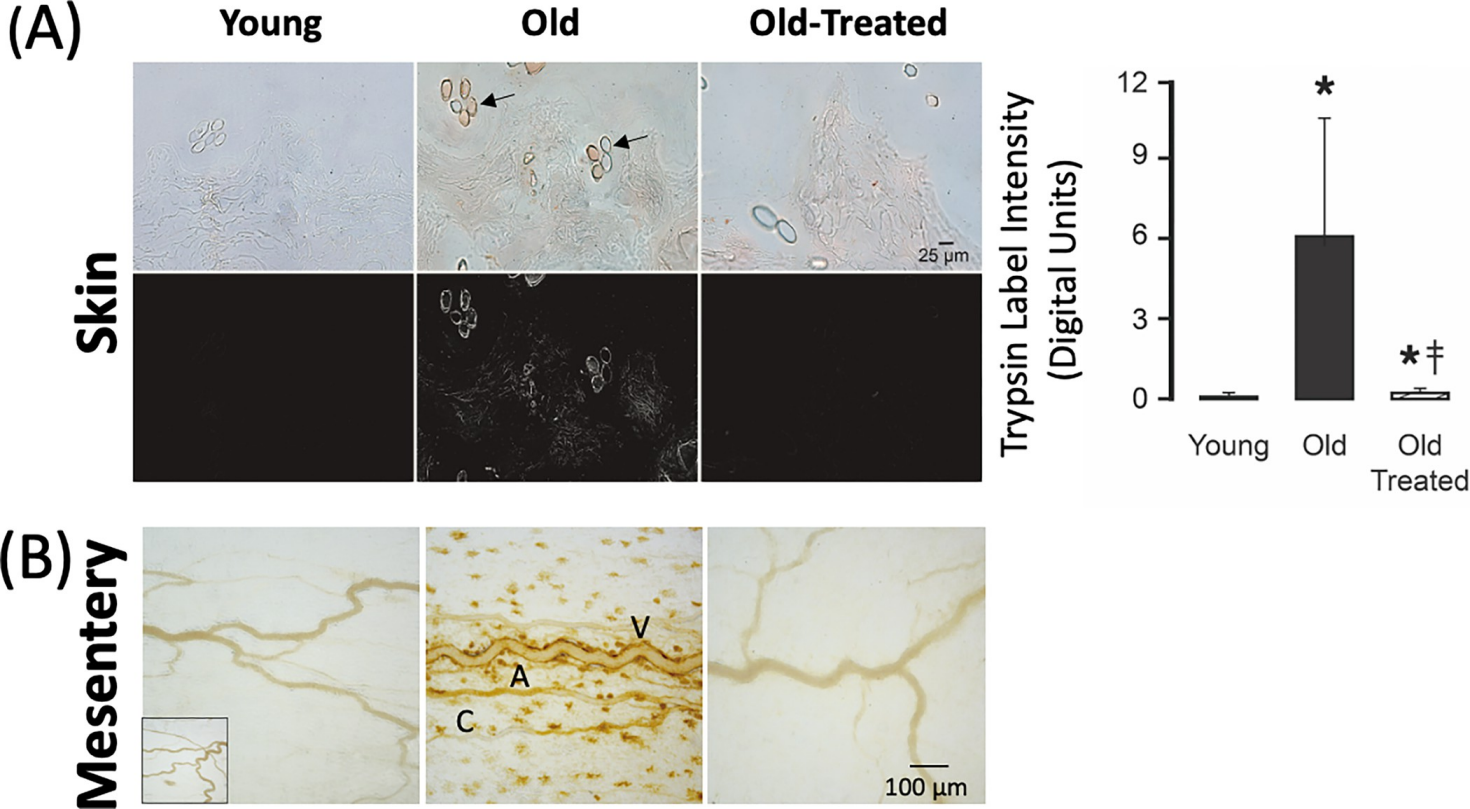
Pancreatic trypsin label density in (A) abdominal skin and (B) in enface view of the mesentery is enhanced in the old. In (B), arterioles (A), venules (V), and capillaries (C). Groups, labeling, and measurement analysis are the same as in Fig 1.

In young rats, all tissues in this study (intestinal wall, mesentery, liver, lung, heart, kidney, brain, skin) (Figs 1–3) exhibit low immunolabel density for pancreatic trypsin. Compared to other tissues of the young, the villi of the small intestine and the lung tissue, have a slightly enhanced trypsin label intensity.

In contrast, the tissues of old rats have a significantly increased trypsin label density (Figs 1–3). High densities are on sections of the intestine, liver, and lung, organs that are in the pathway of digestive enzymes leaking from the small intestine, including the venules of the mesentery (Fig 3B). Trypsin labels are enriched in extracellular spaces (e.g. between heart muscle cells), in the wall of capillaries (e.g. brain) (Fig 2, arrows), and in the follicles of the skin.

**Fig 4:**
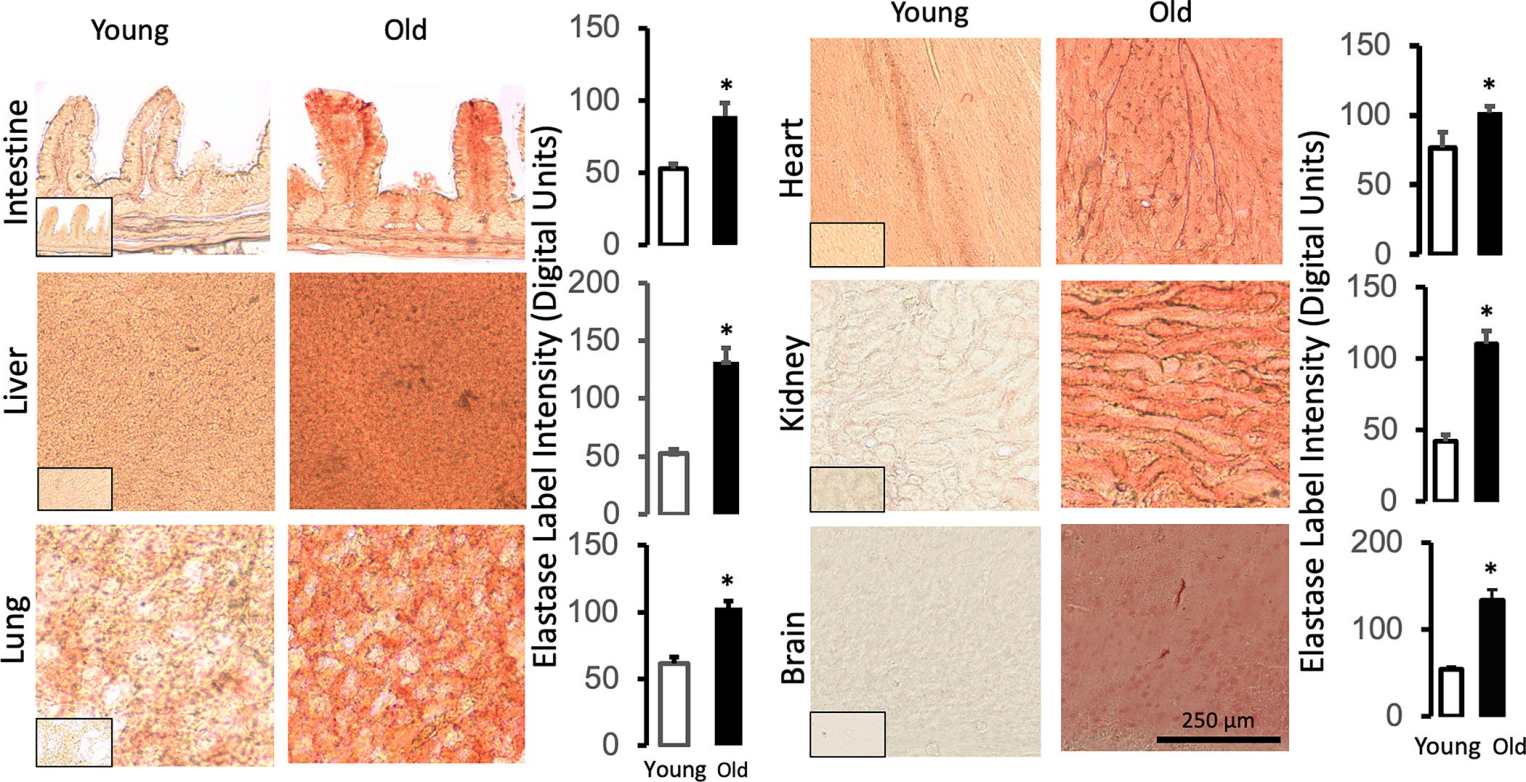
Infiltration of pancreatic digestive elastase into organs of the old but not the young. Sections are labeled with a brown substrate. The inserts show controls without the use of the primary antibody. The histograms (right column) show mean ± SD of the image intensity (in digital units after black and white conversion; not shown). The digital measurements were carried out on single larger tissue sections (~ 4mm x 5mm) by the placement of a digital window (20μm x 30μm) with 30 random measurements per section. Each tissue is relatively uniformly infiltrated by digestive enzymes (on a length scale of > 20μm) as noted by the relatively small standard deviation for the label intensities. The tissues exhibit non-uniform label intensities on a smaller scale. The length scale for all images is the same (shown in the old brain images). *p<0.05 compared with the young group.

**Fig 5:**
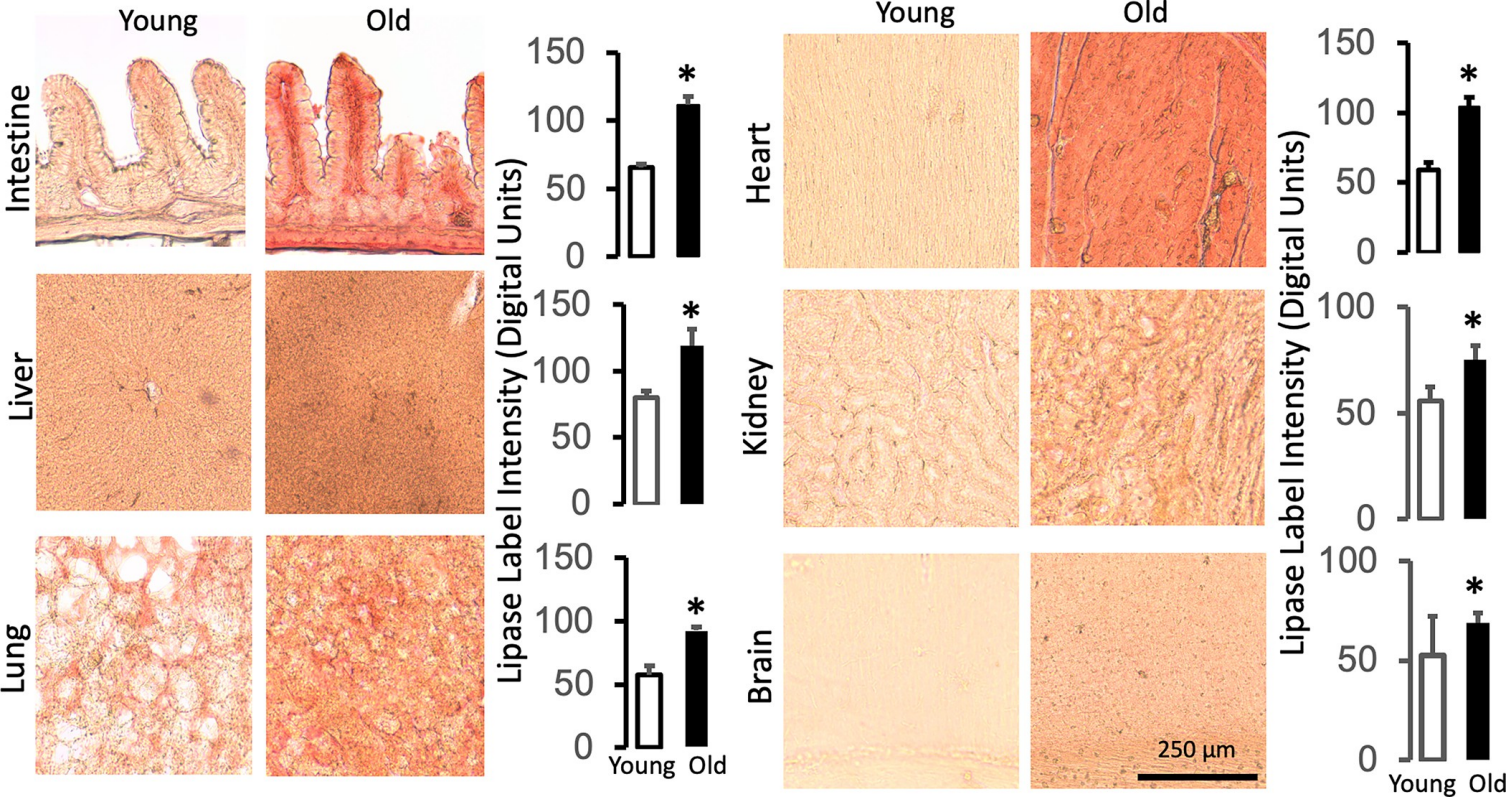
Pancreatic lipase accumulates in the organs of the old. See Fig 4 for immune-labeling and measurement techniques. Sections are labeled with brown substrate. The length scale for all images is the same (shown in the old brain images). *p<0.05 compared with the young.

**Fig 6:**
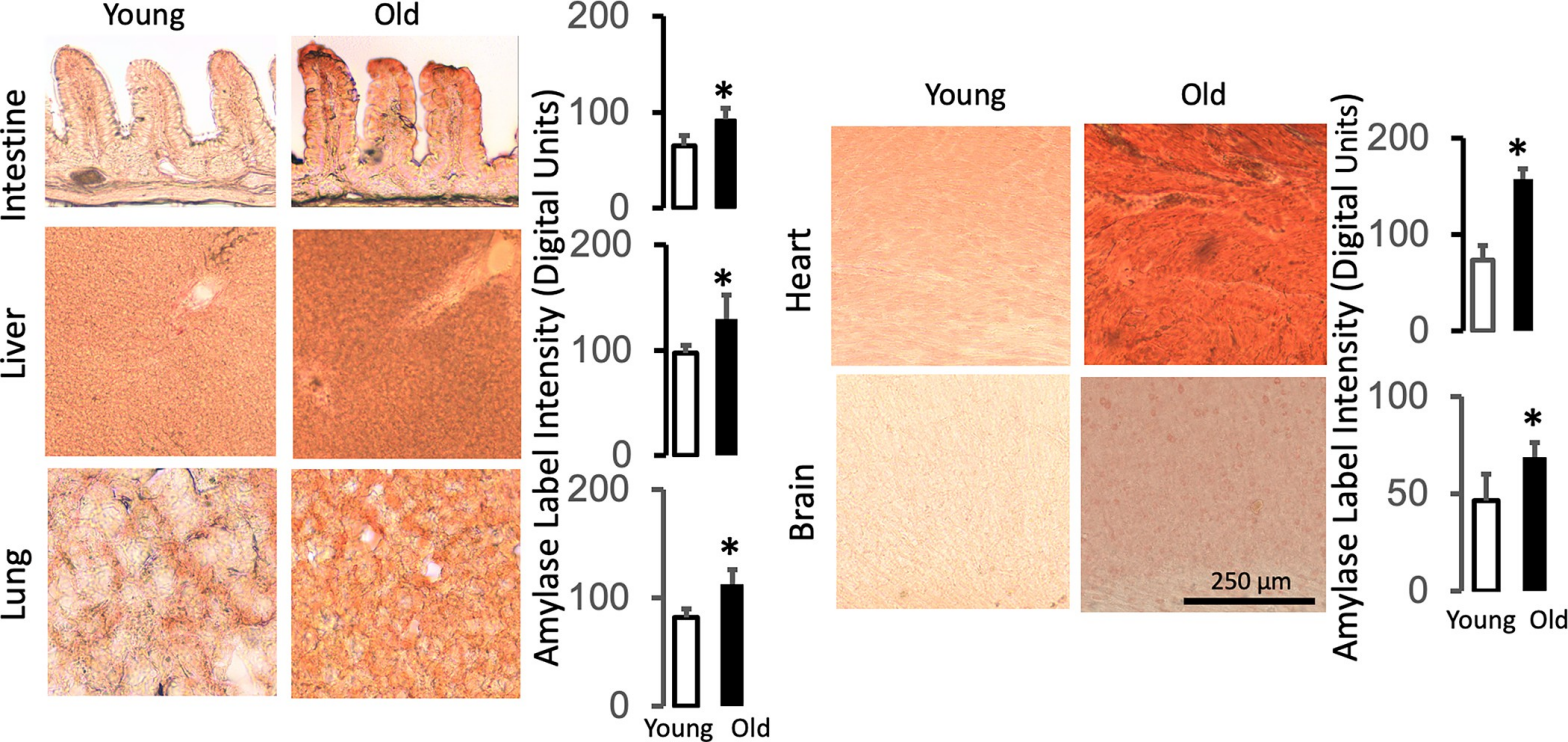
Pancreatic amylase accumulation accumulates in the organs of the old. Immunolabeling techniques and measurements are the same as in Fig 4. The length scale for all images is the same (shown in the old brain images). *p<0.05 compared with the young.

Pancreatic elastase, lipase, and amylase also exhibit low immunolabel densities in young tissues, which is increased in the old (Figs 4–6, respectively). The labeling pattern of the pancreatic enzymes is also tissue-type specific (e.g. with interstitial accumulation between myocytes or in the microvasculature of the brain). However, the average label density is relatively uniform within each old organ as seen by the label density variances (<10%) across individual tissue sections (Figs 4–6, histograms). The measurements suggest that key pancreatic digestive enzymes have uniformly infiltrated the vital organs of old rats outside the pancreas in addition to the villi of the upper jejunal intestine.

Two-week treatment of the old rats with oral small molecular weight trypsin inhibitor significantly reduces the accumulation of trypsin in these vital organs and the skin (Figs 1–3, old treated groups) although not to the low level of the young. An exception is the brain, which exhibits trypsin label density in old treated rats that almost reaches the level in the young (no significant difference) (Fig 2). After the treatment of old rats, we also see low trypsin label densities in the mesentery that are not different from the young (Fig 3B).

### Intestinal mucin-epithelial barrier and digestive enzyme accumulation in the small intestine

**Fig 7:**
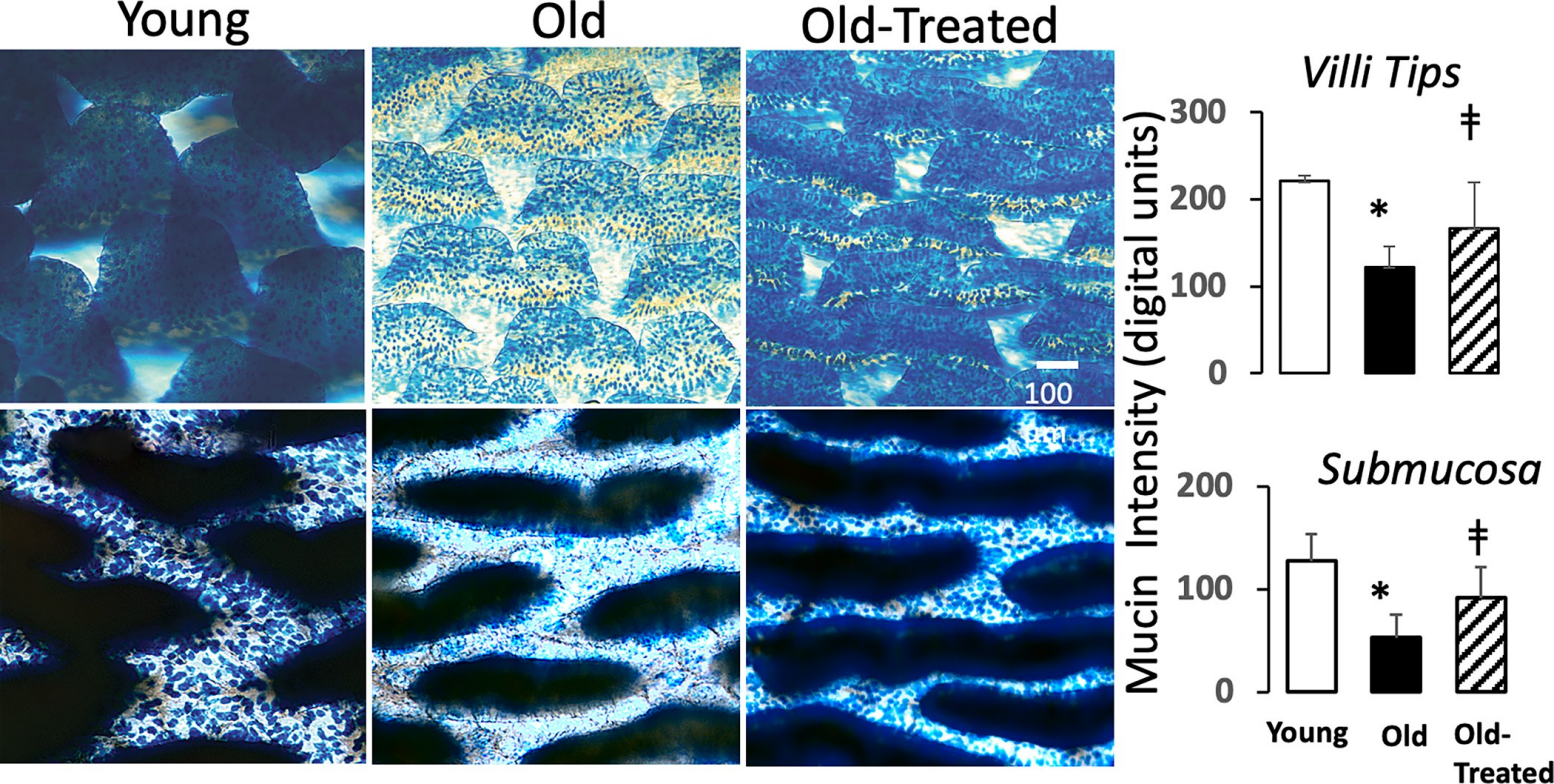
Mucin layer density in the small intestine is reduced in the aged. Enface view of the small intestine (upper jejunum) in young, old, and old-treated rats (same as in Fig 1) after labeling mucin with alcian blue. Histograms show mucin-label intensities at the villi tip and the submucosa. *p<0.05 compared with young group, ‡p<0.05 compared with old untreated rats.

The villi in the upper jejunal segment of the rat small intestine have an elongated crest shape, with an alignment parallel to the long axis of the intestine (Fig 7). The capillary network inside the villi crests is preserved in the old (S1 Fig).

**Fig 8:**
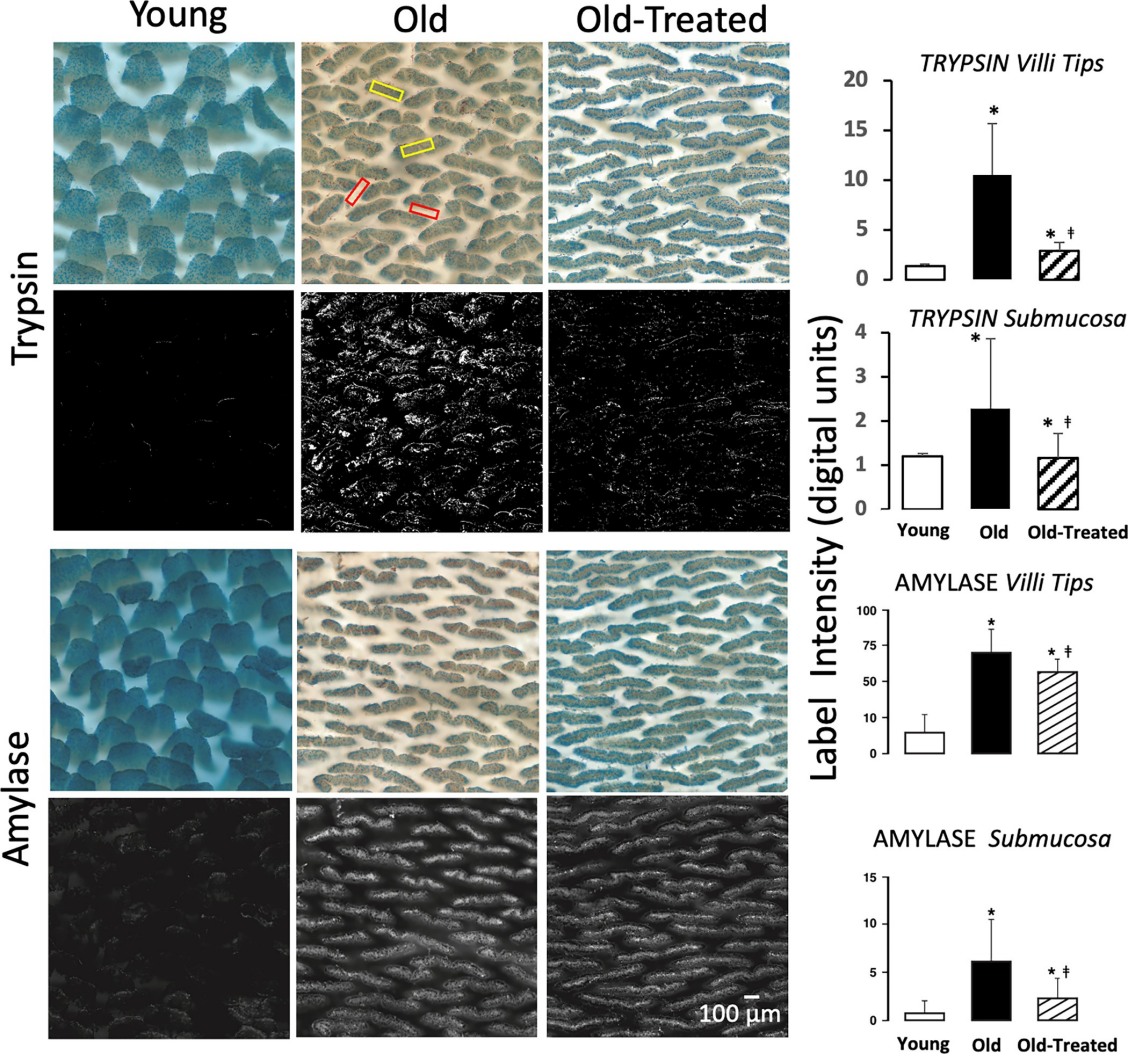
Escape of digestive enzymes across the mucosal barrier in the old with reduced mucin layer. Enface view of small intestine with dual labeling of mucin (blue) and pancreatic trypsin and amylase by immunohistochemistry (brown). Histograms of the enzyme label optical intensity on images after digital color extraction of the brown enzyme labels and conversion into black and white (lower panels). Trypsin and amylase measurements were carried out separately on the villi (yellow windows) and between the villi at the submucosa level (red windows). *p<0.05 compared with young group, ‡p<0.05 compared with old untreated rats.

The mucin layer on the epithelium of the small intestine, a barrier for digestive enzymes [16], has significantly reduced density in the aged, both on and between villi crests (Fig 7). Co-labeling of mucins and pancreatic trypsin or amylase in the intestine demonstrates that the reduced mucin density in the old is accompanied by accumulation of trypsin and amylase in the intestinal wall, both when measured at the villi tips and at the level of the submucosa (Fig 8). The oral trypsin inhibitor treatment partially restores the mucin layer in the old (Fig 7) and attenuates the accumulation of these digestive enzymes in the intestinal wall (Fig 8).

**Fig 9:**
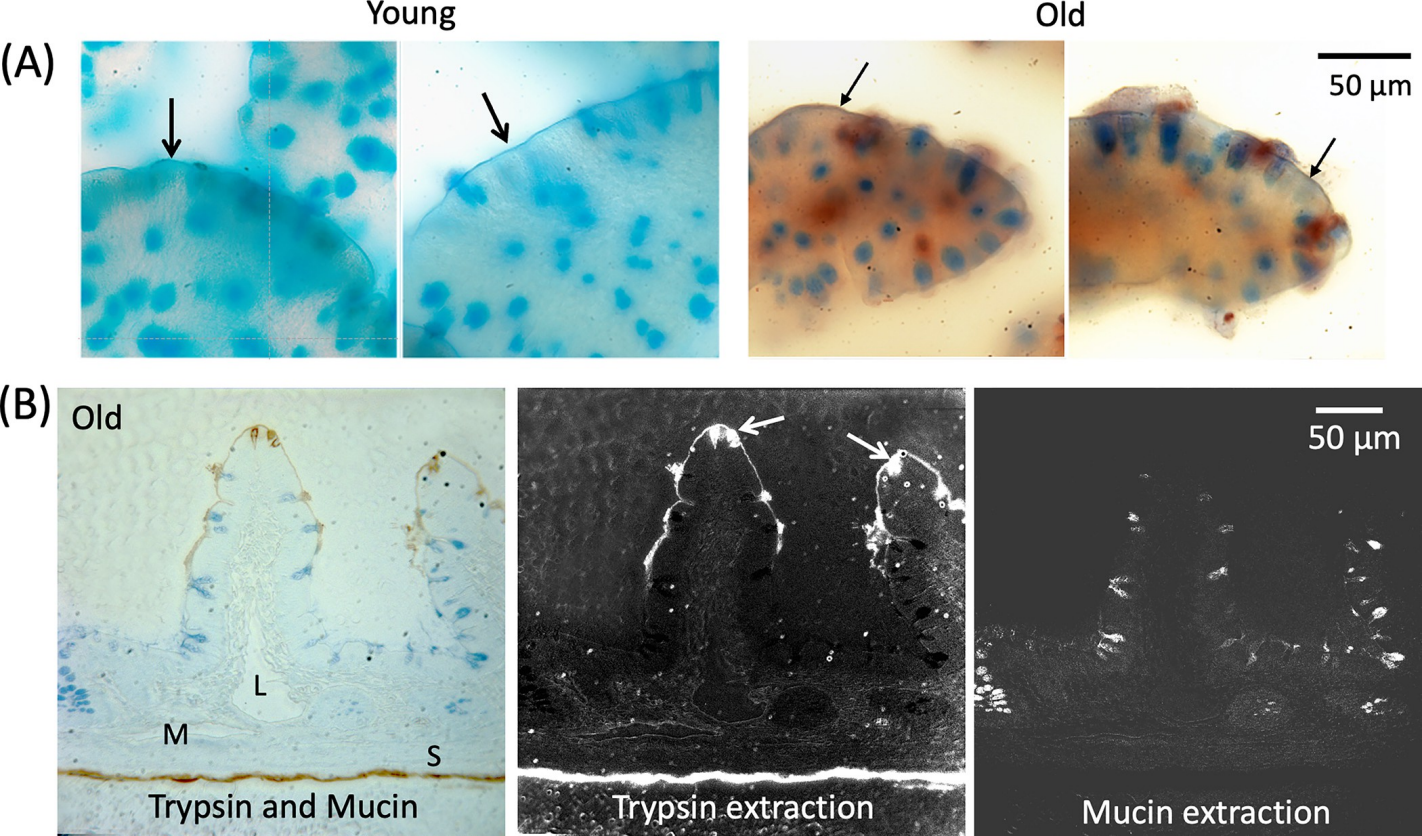
Trypsin leaks at the tip of the intestinal villi. **(A)** Crossection (30 μm thickness) of the small intestine villi from young and old rats with dual staining for mucin (blue) and trypsin (brown). The ubiquitous globular mucin and the mucin attached to the villi tips (thick arrows) in young animals is reduced in the old (thin arrows), accompanied by the entry of trypsin into the villus interstitial space. **(B)** Thin cross-section (1 μm) of intestinal villus of old rat, dual labeled with mucin and trypsin, and shown separately after digital color extraction (middle and right panel). Sites of entry of trypsin (arrows, middle panel) are in goblet cells with depleted mucin (right panel). Traces of trypsin immunolabel are present in the lymphangion (L), and the microvasculature (M) as well as prominently in the intestinal serosa (S).

The loss of mucin in the old includes goblet cell-associated mucin 13 (arrows) on the epithelial brush border and mucin 2 from goblet cells (Fig 9A). Thin crossections of the intestinal villi in the old show that the highest density of trypsin is at their tip, especially inside the residual cavities of goblet cells after mucin discharge, and in the epithelial brush border with reduced mucin label (Fig 9B). Traces of trypsin label is detectable in the lamina propria, the microvasculature, lymphatics, and the intestinal serosa (see also Fig 1).

### Collagen degradation

**Fig 10:**
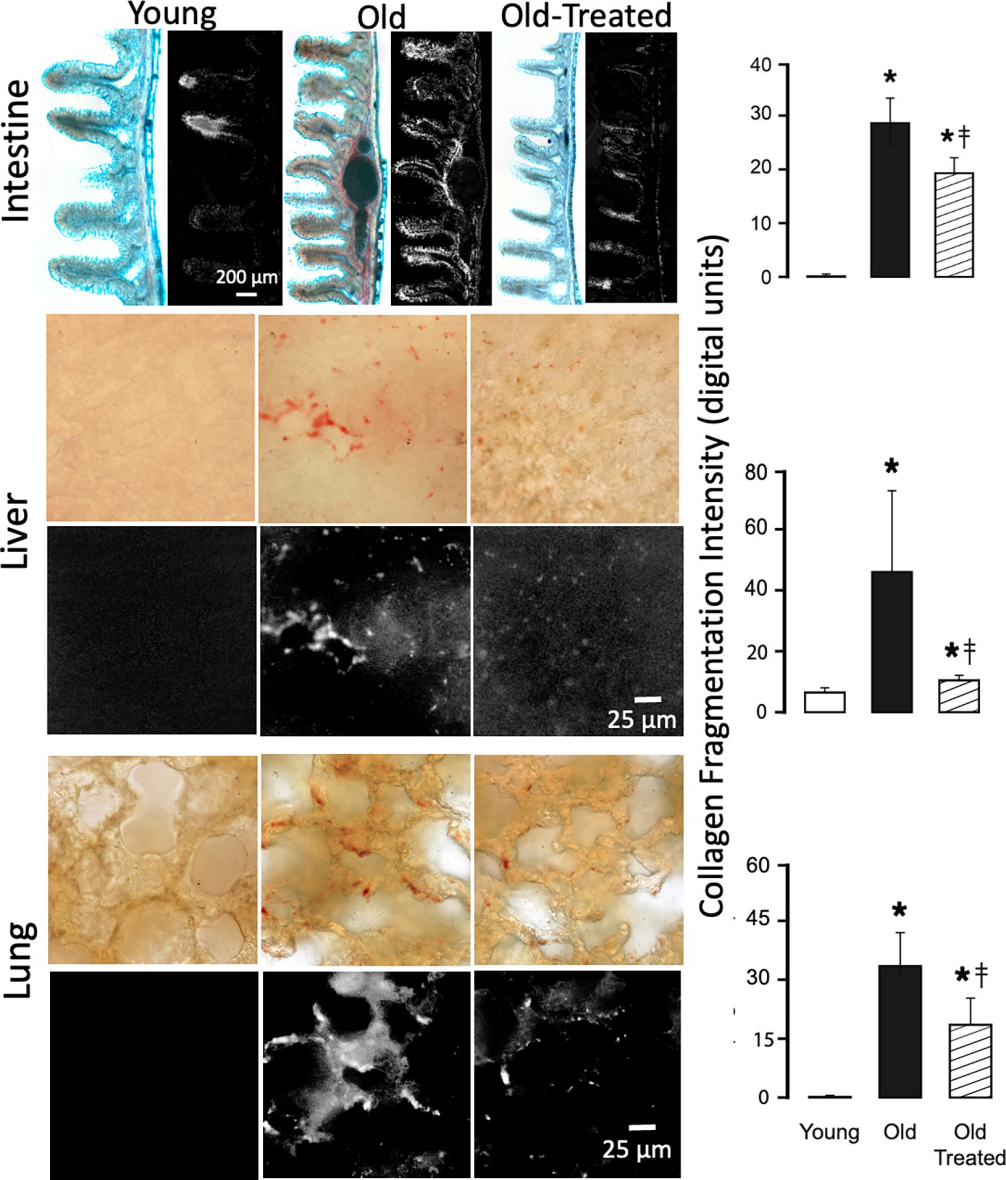
Collagen damage in the old intestine, liver, and lung is higher than in the young and attenuated by the blockade of leaking digestive enzymes. Hybridizing peptides show tissue sites with collagen damage in the old that is significantly elevated compared to the young. Digital color extraction of the red peptide label is shown in the black/white panels and histograms of digital intensity measurements. *p<0.05 compared with the young group, ‡p<0.05 compared with old untreated rats.

**Fig 11:**
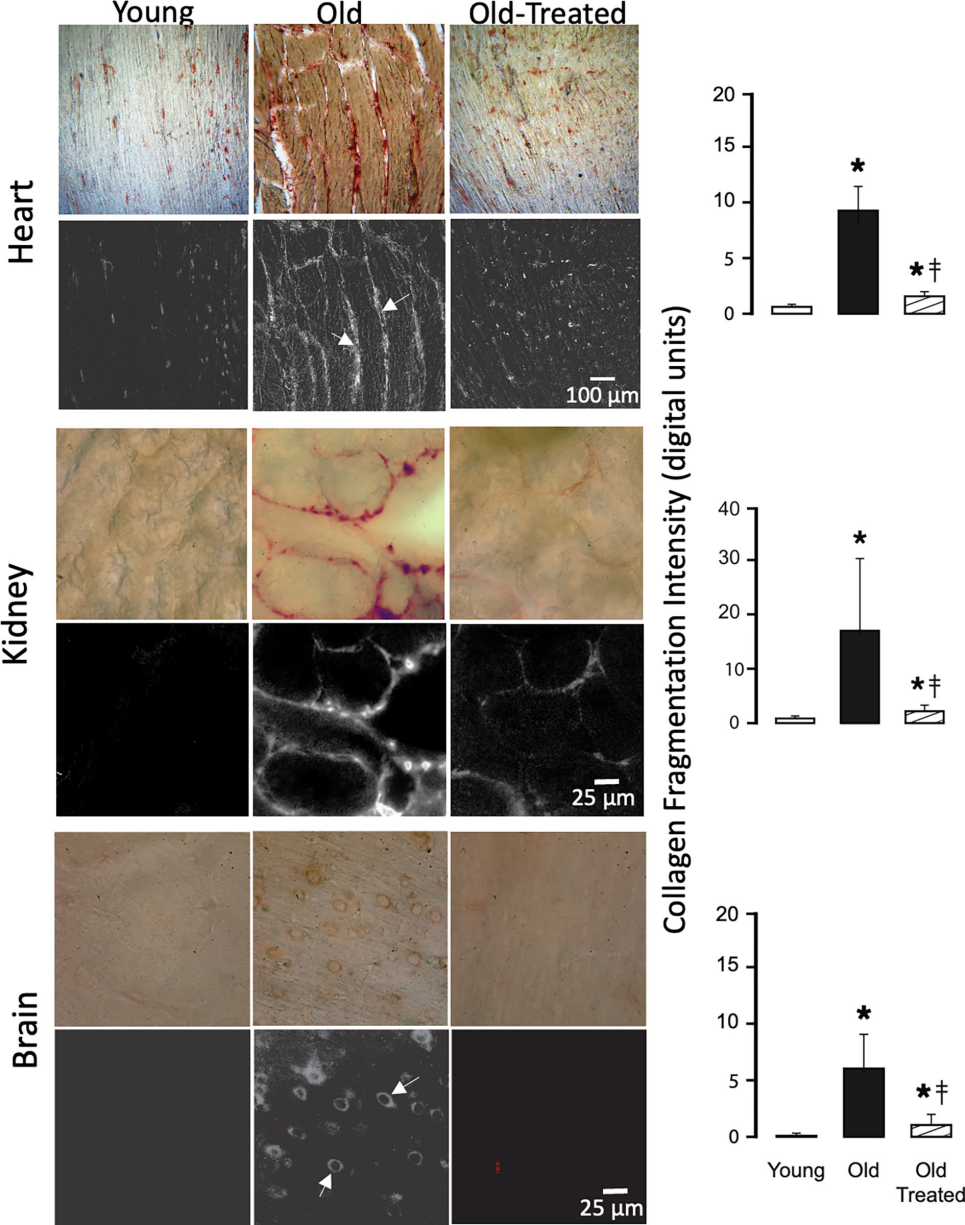
Collagen damage in old heart, kidney, and brain is higher than in the young and attenuated by the blockade of leaking digestive enzymes. Labeling with hybridizing peptides and measurement techniques are the same as in Fig 10. *p<0.05 compared with young group, ‡p<0.05 compared with old untreated rats.

**Fig 12:**
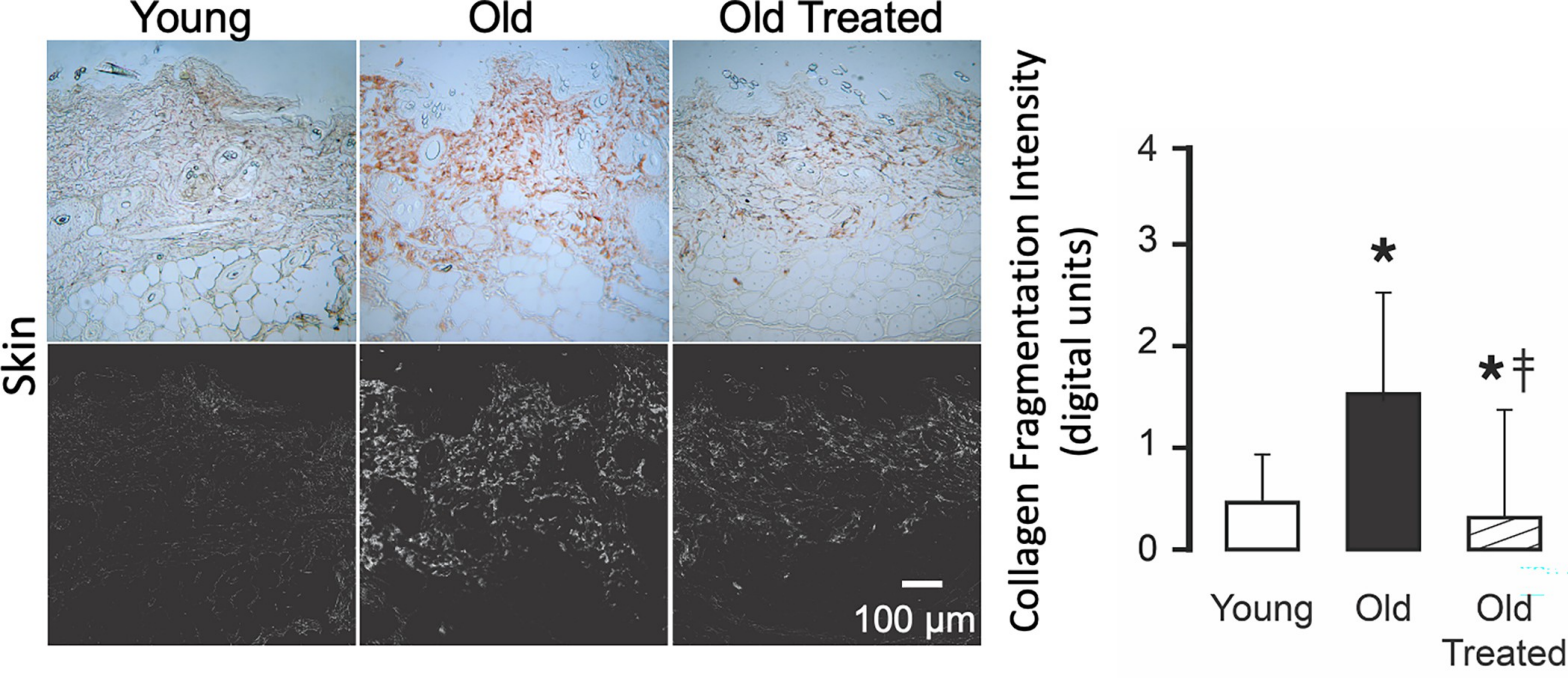
Collagen damage in the skin of the old is higher than in the young and attenuated by the blockade of leaking digestive enzymes. For labeling and measurement techniques see Fig 10. *p<0.05 compared with young group, ‡p<0.05 compared with old untreated rats.

Organs of the young have low levels of collagen damage, detected by hybridizing peptides. In contrast, old organs have uniformly enhanced collagen damage in all organs we studied (Figs 10–12). The damage is significantly reduced by the two-week oral treatment with trypsin inhibitor.

Whereas in the intestine collagen damage is present in the young and the old, more villi in the old exhibit damage. The tips of the villi have the highest collagen damage in both young and old, which coincides with the location where digestive enzymes cross the mucin epithelial barrier (Fig 9B). The old have more villi and larger tissue areas in the lamina propria with collagen damage. The serosa also exhibits collagen damage in young and old at the same location where trypsin has accumulated (Fig 9B).

In the heart muscle, collagen degradation is accompanied by an expansion of the interstitial space between muscle fibers (Fig 11). In the brain, it is diffused throughout the tissue and enhanced in the wall of capillaries (Fig 11). In the skin, collagen damage occurs in all collagen fibers of the epidermis and dermis of the old (Fig 12). Just like in all other organs we studied it is reduced by oral blockade of pancreatic trypsin.

### Insulin receptor cleavage

**Fig 13:**
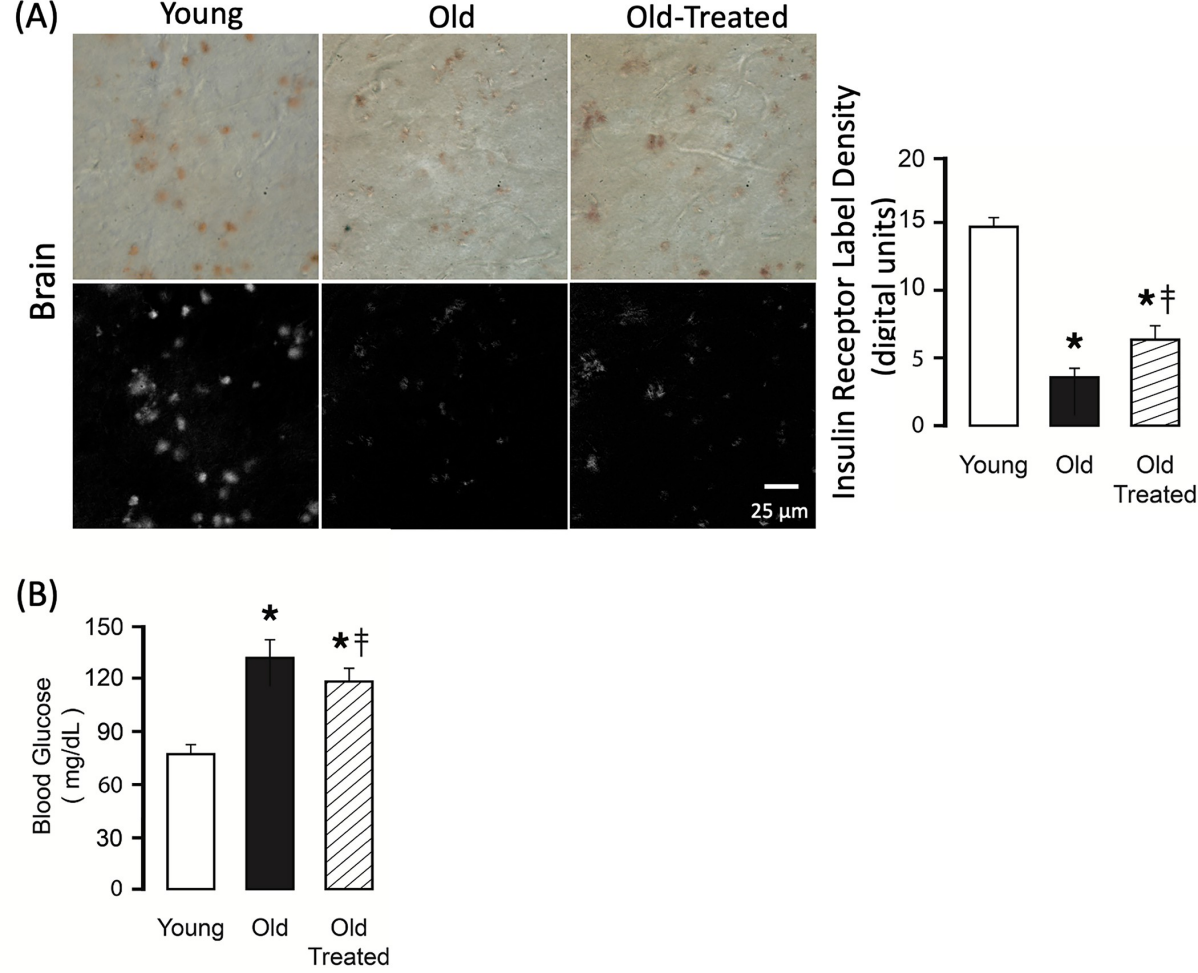
Insulin receptor cleavage and hyperglycemia in the old. **(A)** Immunolabel density of the extracellular domain of the insulin receptor (brown substrate) in sections of the brain cortex of young and old rats without and with temporary trypsin treatment. The top row shows original color images and the bottom row insulin receptor density after digital color extraction of the brown substrate with a histogram of the label density measurements. **(B)** Blood glucose values in the same groups. *p<0.05 compared with young group, ‡p<0.05 compared with old untreated rats.

To determine whether digestive proteases may be involved in membrane receptor cleavage we investigated the insulin receptor in the brain, an organ distant from the intestine. Immunohistochemistry with a monoclonal antibody that binds to the extracellular domain of the insulin receptor [23] shows its distribution in the cerebral cortex of the rat. The density of the insulin receptor ectodomains is significantly reduced in the aged, compared to the young, and in part restored after two-week trypsin inhibition (Fig 13A). It coincides with an increase of plasma glucose levels in the old without but not with the trypsin treatment (Fig 13B).

## Discussion

The current results in the rat bring to light a fundamental mechanism for progressive multi-tissue degradation in the aged that is a consequence of the need to digest. Whereas located in the lumen of the small intestine, we find multiple types of pancreatic digestive enzymes in organs outside the lumen of the intestine of the old and less in the young. Pancreatic enzymes appear even in the brain indicating that they have breached two main barriers, the intestinal epithelial and the blood-brain barrier. As demonstrated in the case of pancreatic trypsin, the enzymes escape across the mucin-epithelial barrier in the small intestine and accumulate in vital organs. The digestive enzymes trigger a hallmark of aging, as detected by the breakdown of collagen, and they generate signatures for insulin resistance in the form of hyperglycemia and extracellular insulin receptor cleavage. A two-week treatment of old rats with trypsin inhibitor restores in part the mucin-epithelial barrier, reduces the trypsin accumulation in vital organs, attenuates the collagen degradation, and restores in part the insulin receptor density and the blood glucose level in the old.

### Digestive enzyme compartmentalization in the gastrointestinal tract

These results are in line with the central role of the gastrointestinal tract and the digestive enzymes in several diseases [24] and multiorgan failure and death [13]. A key requirement for the prevention of the degrading actions of the pancreatic digestive enzymes outside the gastrointestinal tract is their compartmentalization inside the lumen of the pancreatic ducts and small intestine by the mucin/epithelial barrier [25]. This barrier can be breached by multiple mechanisms, including but not limited to the reduction of the oxygen supply [16], the presence of partially digested food constituents [26], and unbound free fatty acids [27]. Even in the young, the tip of the villi is infiltrated by digestive enzymes while also the site for epithelial cell apoptosis [28], suggesting that repeat injury and continuous growth of villi is part of a normal cycle during digestion [29]. However, the current evidence suggests that chronic reconstitution of the intestinal villi is incomplete with reduced mucin density and enhanced digestive enzyme leak (Figs 7–9).

### Transport pathways for digestive enzymes out of the intestine

Once digestive enzymes leak across the epithelium/mucin barrier into the lamina propria of the intestinal villi, three pathways serve to reach the systemic circulation. Digestive enzymes can be carried (a) via the intestinal microcirculation and the portal venous system, (b) via the intestinal and the mesenteric lymphatics [30] and the lymphatic ducts into the venous circulation, bypassing the liver, and (c) across the submucosa, the muscularis, and the serosa of the intestine into the peritoneal fluid [31]. The elevated label densities in the intestine and the liver of old rats for pancreatic lipase, elastase, and amylase, suggest a pathway predominantly via intestinal venules and hepatic portal veins. Other pathways involved in different stages of aging remain to be determined.

Within old organs, all tissue regions have an elevated digestive enzyme label density (Figs 1–6). The density is enhanced in the extracellular space (e.g. between heart muscle fibers; Fig 2), consistent with the fact that as water-soluble proteins without known membrane receptors digestive enzymes have no effective transport mechanisms across intact cell membranes [32].

### Digestive protease activity in aging

Pancreas digestive enzymes that escape out of the small intestine are in an *active* form following conversion from their proform by enterokinases in the duodenum [33]. Their activity in plasma and organs outside the intestine depends, however, on the levels of endogenous inhibitors (e.g. serpins synthesized in the liver) and serve to control digestive enzyme activity. However endogenous inhibitors can be overwhelmed when larger amounts of digestive enzymes pass through the epithelial/mucin barrier into plasma, e.g. in an acutely ischemic intestine [16] or during a postprandial period even in the young [18]. The digestive enzyme *activity*, as a balance between digestive enzymes, breakdown products they produce, and inhibitor concentrations, remains to be determined with in-vivo zymographic techniques in aging.

### Tissue degradation by pancreatic digestive proteases

Digestive enzymes are optimized to degrade most biological tissues. Inside the lumen of the intestine, they are in high concentrations, in an active state, and are relatively non-specific. Pancreatic trypsin, for example, degrades most proteins irrespective of the source and causes cell dysfunctions.

Once digestive proteases have breached the mucin/epithelial barrier they in turn break down the mucin layer [16], cleave the extracellular domain of interepithelial junction proteins (E-cadherin), open the epithelial brush border, and even destroy the villi [15,34]. Upon entry into organs outside the intestine, numerous cell and tissue functions are at risk by active digestive enzymes. Pancreatic trypsin in the circulation triggers the activation of proMMPs [35,36]. The protease activity leads to ectodomain receptor cleavage and the reduction of their cell functions, such as cleavage of the insulin and leptin receptors with associated insulin and leptin resistance [18,23,37]. The extent of surface receptor and glycocalyx cleavage in different organs of the old remains to be investigated and may constitute a mechanism for their spectrum of attenuated cell functions (e.g. protein homeostasis, nutrient sensing, stem cell exhaustion, intercellular communication) [38] and chronic inflammation [39].

A key finding of the current study is the extensive cleavage of collagen in the organs we investigated (Figs 10–12). The breakdown of the collagen structure, detectable with hybridizing peptides binding to fractures in the triple-helical collagen molecule, can be produced either by mechanical stress or by exposure to proteases (e.g. trypsin) [40] and precedes the collagen restructuring or loss of fibers. Collagen damage promotes the disassembly of integrin attachments [41], and integrin-dependent cell behavior [42], and enhances apoptosis [43]. Collagen damage mediated by pancreatic digestive proteases and the secondary enzymes they activate may thus be a central mechanism for biological aging.

The tissue degrading processes by digestive enzymes are in line with the coincidence of chronic diseases (e.g. diabetes) during aging and multiorgan failure at the end of life. Our evidence supports the idea that a slow leak of digestive enzymes out of the gastrointestinal tract may lead to the gradual progression of organ dysfunction in aging [18], whereas a major breach of the mucin-epithelial barrier with a rapid escape of digestive enzymes leads to acute organ failure [34].

### Digestive protease inhibition

The current evidence indicates that the accumulation of digestive enzymes in tissues outside the gastrointestinal tract in the old can be reduced by two-week oral trypsin inhibition. This intervention needs to be nuanced to block autodigestion but not digestion. Even though the trypsin inhibitor in this study was administered orally, the concentration in the drinking water was kept sufficiently low so that a temporary treatment did not lead to a detectable attenuation of digestion, such as a reduction of body weight. The strategy served to restore the mucin layer on the intestinal villi (Fig 7), reduce the leak of digestive enzymes into the intestinal wall (Figs 1, 4 and 8), and accumulation of digestive enzymes in organs outside the intestine (Figs 1–6).

### Aging interventions and autodigestion

In multiple species, caloric reduction without starvation or timed eating attenuates age-associated morbidities [44,45]. The autodigestion hypothesis may provide an insight for such a benefit. A single meal can be accompanied by an instantaneous leakage of digestive enzymes into the central circulation within less than an hour of food consumption [18]. Reduction in the daily frequency and the volume of food passing through the small intestine may attenuate damage to the mucin/epithelial barrier and consequently reduce enzyme leak. In light of the continuous repair of the intestinal epithelium [46], prolonging the periods between meals may enhance the reconstitution of the microvilli and the epithelial/mucin barrier and thereby minimize autodigestion. The exact chronology of intestinal damage by leaking digestive enzymes and repair of the villi with their mucin/epithelial barrier during and after a meal remains to be elucidated.

## Supporting information

S1 Fig Brightfield images of typical capillary networks in villi of the upper jejunum in young and old rats. The capillaries are filled with carbon solution.
(TIF)
